# Notoginsenoside R1 Protects Against Diabetic Cardiomyopathy Through Activating Estrogen Receptor α and Its Downstream Signaling

**DOI:** 10.3389/fphar.2018.01227

**Published:** 2018-11-02

**Authors:** Bin Zhang, Jingyi Zhang, Chenyang Zhang, Xuelian Zhang, Jingxue Ye, Shihuan Kuang, Guibo Sun, Xiaobo Sun

**Affiliations:** ^1^Institute of Medicinal Plant Development, Peking Union Medical College and Chinese Academy of Medical Sciences, Beijing, China; ^2^Key Laboratory of Bioactive Substances and Resources Utilization of Chinese Herbal Medicine, Ministry of Education, Beijing, China; ^3^Beijing Key Laboratory of Innovative Drug Discovery of Traditional Chinese Medicine (Natural Medicine) and Translational Medicine, Beijing, China; ^4^Key Laboratory of Efficacy Evaluation of Chinese Medicine Against Glyeolipid Metabolism Disorder Disease, State Administration of Traditional Chinese Medicine, Beijing, China; ^5^Department of Animal Sciences, Purdue University, West Lafayette, IN, United States

**Keywords:** diabetes mellitus, diabetic cardiomyopathy, estrogen receptor, apoptosis, oxidative stress

## Abstract

Diabetic cardiomyopathy (DCM) leads to heart failure and death in diabetic patients, no effective treatment is available. Notoginsenoside R1 (NGR1) is a novel saponin that is derived from Panax notoginseng and our previous studies have showed cardioprotective and neuroprotective effects of NGR1. However, its role in protecting against DCM remains unexplored. Herein, we examine potential effects of NGR1 on cardiac function of diabetic db/db mice and H9c2 cardiomyocytes treated by advanced glycation end products (AGEs). *In vitro* experiments revealed that pretreatment with NGR1 significantly decreased AGEs-induced mitochondria injury, limited an increase in ROS, and reduced apoptosis in H9c2 cells. NGR1 eliminated ROS by promoting estrogen receptor α expression, which subsequently activated Akt and Nrf2-mediated anti-oxidant enzymes. *In vivo* investigation demonstrated that NGR1 significantly reduced serum lipid levels, insulin resistance, the expression of enzymes related to cardiomyopathy, and the expression of apoptotic proteins. Finally, NGR1 improved cardiac dysfunction and attenuated histological abnormalities, as evidenced by elevating ejection fraction and fractional shortening, and reducing cardiac fibrosis. Mechanistically, NGR1 promoted ERα expression, which led to the activation of Akt-Nrf2 signaling and the inhibition of the TGFβ pathway. Collectively, these results strongly indicate that NGR1 exerts cardioprotective effects against DCM through its inhibition of oxidative stress and apoptosis, and eventually suppresses cardiac fibrosis and hypertrophy, which suggests that NGR1 is a potential therapeutic medicine for the treatment of DCM.

## Introduction

Diabetic complications have become a more severe threat to human health in recent years. Cardiovascular complications, including diabetic cardiomyopathy (DCM), account for approximately 80% of diabetic deaths (Amos et al., 1997). Pathologically, diabetes induces cardiac dysfunction with no evidence of hypertension and coronary artery disease, and is characterized by myocardial insulin resistance, cardiac fibrosis, ventricular hypertrophy and heart failure (Liu et al., 2015). Accumulating research has demonstrated underlying disease mechanisms that include oxidative stress, inflammation, mitochondrial dysfunction, and lipotoxicity; each potentially contributing to the pathophysiology of DCM (Cai et al., 2006; Kuwabara et al., 2015; Cheng X. et al., 2016; Vulesevic et al., 2016). Although substantial progress has been made in the investigation of DCM in recent years, the underlying molecular mechanisms of DCM are still not fully understood. Beyond this, there are currently no disease-specific drugs to treat DCM in the clinic. Thus, it is important to clarify its molecular mechanisms and to search for potential compounds that provide protection against DCM.

Continuous hyperglycemia and abnormality of lipids in diabetic hearts induce oxidative stress, which results from an imbalance between reactive oxygen species (ROS) and/or reactive nitrogen species (RNS) generation and their clearance (Cai and Kang, 2001). ROS contain one or more unpaired electrons, making them susceptible to interaction with biological molecules such as DNA, carbohydrates, and proteins. In the DNA, ROS can interact with both the bases and the sugar residues of the nucleic acid sequence. ROS-related oxidation of DNA is one of the main causes of mutations, such as non-conventional single-strand breaks and intra- or interstrand DNA crosslinks (Dickinson and Chang, 2011). Moreover, ROS interact with carbohydrates, and the subsequent oxidized carbohydrates can glycate proteins and form advanced glycation end products (AGEs). The mechanism by which AGEs induce damage is through a process called crosslinking (proteins can be covalently bonded and form chains), which causes intracellular damage and apoptosis (Simm et al., 2007). Once formed, ROS are released from cardiac cells and lead to severe inflammation (Bryan et al., 2012). Accumulation of ROS can not only impair cardiac stem cells (Li et al., 2011), but induce collagen expression in atrial fibroblasts through transforming growth factor β1 (Tsai et al., 2011). Thus, to inhibit cardiac oxidative stress might be a potential route for drug development to fight against DCM.

Nuclear factor-erythroid 2-related factor 2/antioxidant responsive element (Nrf2/ARE) signaling plays an indispensible role in controlling transcriptional regulation of the genes encoding endogenous antioxidant enzymes. Phase II antioxidant enzymes include heme oxygenase-1 (HO-1), NAD(P)H quinone oxidoreductase-1 (NQO-1), and γ-glutamylcysteine synthetase heavy subunit (γ- GCS). Nrf2-mediated antioxidant enzymes were reported to be promising therapeutic targets for limiting oxidative stress and promoting cardioprotection (Tsai et al., 2017). Accordingly, discerning how to promote the translocation of Nrf2 and subsequently increase these antioxidant enzymes has become a promising approach in the treatment of DCM.

**FIGURE 1:**
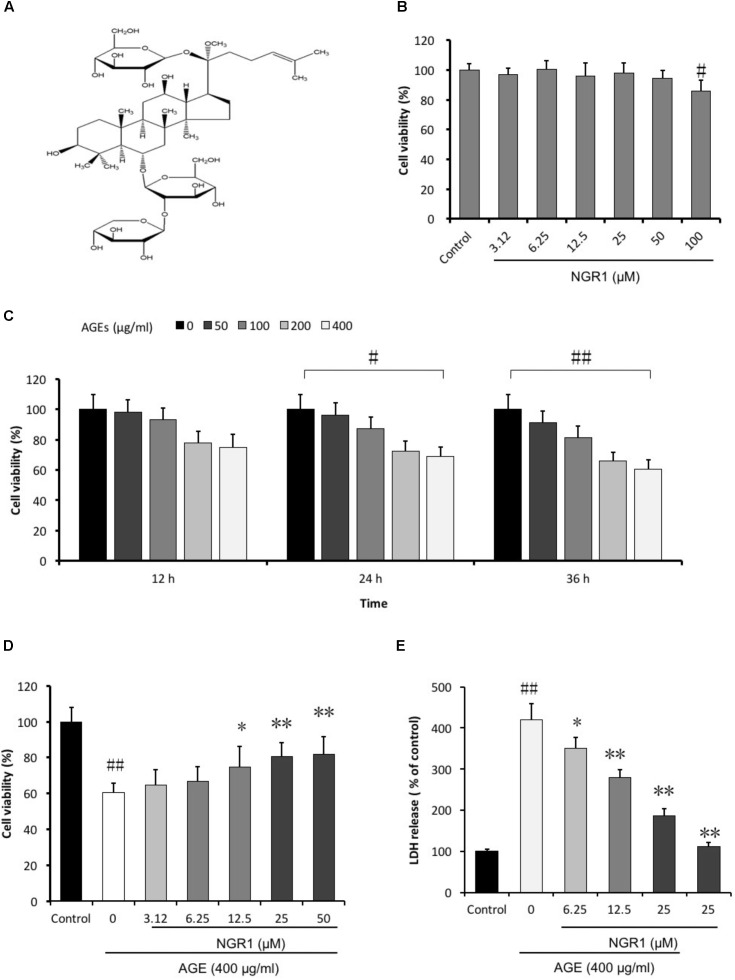
NGR1 protects against H9c2 cell death induced by AGEs. **(A)** Molecular structure of NGR1. **(B)** Toxic effect of NGR1 on H9c2 cell viability was insignificant up to 50 μM. **(C)** H9c2 cell viability was affected by the incubation of AGEs for 36 h. **(D)** NGR1 attenuated H9c2 cell death induced by AGEs. **(E)** NGR1 decreased H9c2 cell LDH release induced by AGEs. The quantitative data are presented as the mean ± SD of three independent experiments. ^#^*P* < 0.05 or ^##^*P* < 0.01 vs. the Control group, ^∗^*P* < 0.05 or ^∗∗^*P* < 0.01 vs. the AGEs group.

Notoginsenoside R1 (NGR1, Figure 1A) is a novel phytoestrogen isolated from *Panax notoginseng* (Burk.) F. H. Chen, an ancient medicinal plant in China that has been reported to treat cardiovascular and cerebral vascular diseases (Son et al., 2009; Liu et al., 2017). Increasing evidence suggests that NGR1 possesses various biological properties including inhibiting cancer metastasis, inflammation, and apoptosis (Zhang and Wang, 2006; Zhong et al., 2015; Lee et al., 2017). Our previous studies have reported that NGR1 exerts its neuroprotective role in both H_2_O_2_-induced oxidative damage and amyloid β25–35-induced neurotoxicity in PC12 cells by blockage of the oxidative stress, apoptosis, and stress-activated MAPK signaling pathways (Ma et al., 2014). Additionally, NGR1 protects against ischemia/reperfusion injuries by regulating oxidative stress- and endoplasmic reticulum stress-related signaling pathways (Yu et al., 2016). However, the protective effect of NGR1 on DCM has not yet been investigated and related molecular mechanisms remain unclear.

Thus, the present study aimed to investigate the protective effects of NGR1 and the molecular mechanisms underlying its effects on H9c2 cells subjected to AGEs- induced injury. In addition, *in vitro* and *in vivo* (db/db mice) studies were undertaken to examine whether NGR1 acts through estrogen receptor (ER)α-dependent activation of the AKT pathway and inhibition of TGFβ signaling, providing the rationale for NGR1 being a therapeutic candidate that can attenuate the development of DCM.

## Materials and Methods

### Reagents

Notoginsenoside R1 (NGR1, molecular weight = 933.14, purity > 98.6) was obtained from Shanghai Winherb Medical S&T Development (Shanghai, China). Metformin (Sino-American Shanghai Squibb Pharmaceuticals Ltd.,) was used as the positive control in this study. All cell culture materials, Dulbecco’s Modified Eagle’s medium (DMEM), fetal bovine serum (FBS), and penicillin/streptomycin were obtained from Gibco (NY, United States). The kits for determining the CK-MB, LDH, and AST enzyme were purchased from Jiancheng Bioengineering Institute (Nanjing, China). Estrogen receptor inhibitor (ICI182780) was obtained from Abcam Co. (Cambridge, United Kingdom).

### Preparation of AGE-BSA

The AGE-BSA was prepared according to the protocol of Zhang et al. (2017). Briefly, 0.07 g bovine serum albumin in PBS was incubated with 0.7926 g D-glucose at 37°C for 8 weeks. Control albumin was incubated without glucose. Endotoxin was removed by Pierce endotoxin removing gel and was determined by ToxinSensor^TM^ chromogenic LAL Endotoxin Assay Kit (GenScript, Piscataway, NJ, United States), which was less than 500 U/L.

### Cell Culture and Treatment

Rat embryonic cardiomyoblast-derived H9c2 cardiomyocytes were purchased from the Cell Bank of the Chinese Academy of Sciences (Shanghai, China) and cultured in DMEM (Gibco, glucose content, 5.5 mM), supplemented with 10% fetal bovine serum, 1% penicillin/streptomycin in a 5% CO_2_ atmosphere at 37°C. For all experiments, the cells were plated at an appropriate density according to the experimental design and were grown for 24 h to reach 70–80% confluence before experimentation. The cells were divided into the following groups: (1) BSA group (Control); (2) BSA + NGR1 group (NGR1); (3) AGE-BSA group (AGEs); (4) AGE-BSA + NGR1 (AGEs + NGR1). H9c2 cells were placed in 96-well plates at 8 × 10^3^ per well for 24 h. The cells were first treated for 12, 24, and 36 h with various concentrations of AGEs.

### Determination of Cell Viability

The MTT assay was employed to determine the cell viability of the H9c2 cardiomyocytes. Cells cultured in 96-well plates (8 × 10^3^ cells/well) were incubated with MTT solution (1 mg/mL final concentration) at 37°C for 4 h after the different treatments. The formazan crystals were dissolved with dimethyl sulfoxide (DMSO, 150 μL/well), and the absorbance was detected at 570 nm on a microplate reader. Cell viability was expressed as the percentage of MTT reduction compared with the control conditions.

### Detection of Mitochondrial Superoxide (ROS)

MitoSOX Red (Molecular Probes), a mitochondrial superoxide indicator, was employed to detect mitochondrial superoxide production in H9c2 cells as described previously (Xie et al., 2015). Briefly, after treatment, the cells were washed once with PBS and incubated with MitoSOX Red (5 μM) in the dark at 37°C for 10 min. The cells were washed with PBS and then observed with a fluorescence microscope. The fluorescence of MitoSOX Red was detected on a microplate reader at the excitation and emission wavelengths of 510 and 580 nm, respectively. As well, the fluorescence was analyzed by flow cytometry (BD Biosciences, United States).

### Mitochondrial Transmembrane Potential (ΔΨm)

JC-1 (5,5′,6,6′-Tetrachloro-1,1′,3,3′-tetraethyl-imidacarbocyanine iodide, Invitrogen, United States) was used to determine the changes in mitochondrial transmembrane potential as previous reported (Rani et al., 2016). Briefly, after treatment, H9c2 cardiomyocytes were incubated with JC-1 (5 μmol/L) in the dark at 37°C for 30 min and were then washed with PBS followed by fluorescence microscopy (DM4000B, Leica, Germany).

### Terminal Deoxynucleotidyl Transferase-Mediated dUTP Nick End Labeling (TUNEL) Staining

Apoptotic H9c2 cardiomyocytes were visualized by TUNEL staining according to the manufacturer’s instructions (Biovision, Milpitas, CA, United States). Briefly, H9c2 cells were cultured in 24-well plates for 24 h. After treatment, the cells were fixed with 1% paraformaldehyde for 15 min. After two washes with PBS, the cells were incubated in the DNA labeling solution for 60 min at 37°C, and then incubated with anti-BrdU- FITC antibody solution in the dark for 30 min. Images were captured using a fluorescence microscope, and the apoptotic cells were counted with at least 100 cells from five randomly selected fields in each group.

### Flow Cytometric Detection of Apoptosis

After the cardiomyocytes cells were treated with AGEs, apoptosis was also determined using an annexin V-FITC/PI Apoptosis kit according to the manufacturer’s instructions (Invitrogen, United States). In brief, the H9c2 cells were harvested, washed twice with cold PBS, incubated with the 5 μL FITC-annexin V and 1 μL PI working solution (100 μg/mL) for 30 min in the dark at room temperature, and cellular fluorescence was measured by flow cytometry analysis.

### Analysis of Caspase-3 and Caspase-9 Activities

Caspase-3 and caspase-9 activities were evaluated with Fluorometric Assay Kits (BioVision, United States) according to the manufacturer’s instructions. Briefly, the cells were resuspended in lysis buffer and kept on ice for 10 min. Then, 50 μL of 2X reaction buffer containing 10 mM dithiothreitol was added to each sample. 5 μL of 1 mM substrate (DEVD-AFC or LEHD-AFC for caspase-3 or caspase-9, respectively) was added and incubated at 37°C for 1.5 h. The samples were read on a fluorometer at 400 nm excitation and 505 nm emission wavelengths. The fold-increases in caspase activities were determined by comparing the results with the level of the control group.

### Animals

db/db mice were used in our studies as they are characterized by metabolic disturbances resembling human Type 2 diabetes mellitus and exhibit a cardiomyopathy with decreased diastolic and contractile performance and altered cardiac metabolism (Hummel et al., 1966; Belke et al., 2000; Aasum et al., 2002). Both female C57BL/KsJ db/db mice (db/db mice) and C57BL/6J mice (6–8 weeks old) were purchased from Shanghai Slac Laboratory Animal Co. Ltd. (Shanghai, China). The mice were maintained under standard laboratory conditions (room temperature at 22°C, humidity of 60% with a 12 h light/dark cycle) and fed with a standard pellet diet and water *ad libitum*. All animal experiments were approved by the Experimental Laboratory Animal Committee of Chinese Academy of Medical Sciences and Peking Union Medical College and performed in accordance with the guidelines of the National Institutes of Health Guide for the Care and Use of Laboratory Animals published by the United States National Institutes of Health (NIH Publication No. 85-23, revised 1996). All sacrifices were performed under pentobarbitone anesthesia, and every effort was made to minimize animal suffering.

After 2 weeks of adaptation, tail blood glucose levels were measured using a glucometer (Roche). db/db mice with fasting-blood glucose >200 mg/dL were considered diabetic and were used for the further experimentation. The mice were randomly divided into six groups (*n* = 8): (1) C57BL/6 group (Control); (2) db/db group (Model); (3) db/db + metformin200 mg/kg/day group (Met); (4) db/db + NGR1 7.5 mg/kg/day group (Low); (5) db/db + NGR1 15 mg/kg/day group (Intermediate); (6) db/db + NGR1 30 mg/kg/day group (High). NGR1 or metformin was fed to the mice by gavage every day for 20 weeks. Mice in Control and Model groups were gavaged with vehicle.

### Fasting Blood Glucose, Serum TCH and TG Levels

Following 20 weeks of treatment, and after overnight fast, fasting blood glucose (FBG) levels and oral glucose tolerance were assessed. The animals were sacrificed by cervical dislocation. Before sacrificing, the blood samples were collected from retro-orbital venous plexus for serum total cholesterol (TCH) and triacylglycerol (TG) determination by the biochemical kits (BioSino Bio-Technology & Science Inc.).

### Echocardiography

M-mode echocardiography was performed using Vevo 770^TM^ High Resolution Imaging System (VisualSonics Inc., Canada) as previously described (Gao et al., 2012). After 20 weeks’ treatment with NGR1 or metformin the mice were anesthetized with abdominal injection of avertin (2,2,2- tribromoethanol, prepared as a 1.2% solution and used in mice at a dosage of 0.25 mL/10 g body weight). The chests of the mice were shaved, which were then placed in the recumbent position. Left ventricle (LV) internal diameter in systole (LVIDs) and diastole (LVIDd), LV posterior wall thickness in systole (LVPWs) and diastole (LVPWd) were determined by M-mode echocardiography. LV end-diastolic volume (LVVd), LV end-systolic volume (LVVs), fractional shortening (FS), ejection fraction (EF), and LV Mass (AW) were automatically calculated by an ultrasound machine.

### Heart Histopathological Examination

At the end of the experiment, hearts of the mice were excised, the left ventricles were fixed in 4% buffered paraformaldehyde for more than 48 h and embedded in paraffin blocks, sectioned, stained with hematoxylin and eosin or Masson stain, and examined using a light microscope by a pathologist who was blinded to the groups under study.

### Myocardial Enzymes Activities

Serum myocardial enzyme activities of lactate dehydrogenase (LDH), creatine kinase MB (CK-MB) and aspartate transaminase (AST) were measured with corresponding detection kits according to the manufacturer’s instructions (Nanjing Jiancheng Bioengineering, China).

### Immunohistochemistry

Immunohistochemical staining was performed as previously (Xu et al., 2016). Briefly, tissue sections were dewaxed then incubated with 1× target retrieval solution (Dako, Carpinteria, CA, United States) for antigen retrieval, followed by 3% hydrogen peroxide and 5% bovine serum albumin for 30 min, respectively. These sections were incubated with primary antibodies overnight at 4°C. Primary antibodies included anti-TGFβ1 (Santa Cruz Biotechnology, Santa, CA, United States) at 1:100 dilution, anti-HO-1 (Abcam, Cambridge, MA, United States) at 1:100 dilution, anti-cleaved caspase-3 (Abcam, Cambridge, MA, United States) at 1:100 dilution, and anti-Collagen I (Abcam, Cambridge, MA, United States) at 1:100 dilution. Secondary antibodies (1:300–400 dilutions with PBS) were incubated for 1 h in room temperature. Sections were then treated with peroxidase substrate DAB (3, 3-Diaminobenzidine, Vector Laboratories, Burlingame, CA, United States) for coloration and counterstained with hematoxylin.

### Transmission Electron Microscope

Hearts were cut into approximately 1 mm^3^ pieces, fixed in 2.5% glutaraldehyde in 0.1 mol/L sodium phosphate buffer (pH 7.4) overnight at 4°C, and osmicated in 1% osmium tetroxide for 1 h at 4°C. Then, the specimens were embedded in a plastic resin and were sectioned at 70 nm with a Reichert E ultramicrotome (Reichert Jung, Vienna, Austria). Uranyl acetate and lead citrate were used for staining of the sections, before examination with a New Bio-TEM H-7500 (Hitachi, Tokyo, Japan).

### Western Blot Analysis

Cytoplasmic and nuclear protein samples were separated by protein extraction kits containing 1% phenylmethylsulfonyl fluoride (CoWin Bioscience Co., Ltd., Beijing, China). Western blots were performed using a standard blotting protocol, as described previously (Zhou et al., 2017). Equal amounts of protein fractions were separated by electrophoresis on 10% SDS-PAGE gels, and transferred into nitrocellulose membranes. Antibody binding to the membranes was visualized by enhanced chemiluminescence. The primary antibodies and second antibodies were listed in Supplementary Table S1.

### Statistical Analysis

Data from at least three independent experiments were expressed as the means ± standard deviation (SD). Statistical comparisons between different groups were measured by one-way ANOVA followed by the Student–Newman–Keuls test. The level of significance was set at *P* < 0.05.

## Results

### NGR1 Protected H9c2 Cardiomyocytes From AGEs-Induced Cell Death

To investigate the protective effects of NGR1 on AGEs-induced H9c2 cardiomyocyte death, we initially evaluated the general toxicity of AGEs. Firstly, the cytotoxic effect of the agent on H9c2 cells were measured. After treatment with various doses (3.12–100 μM) of NGR1 for 24 h, there was no significant difference in cell viability between the groups with low concentrations of NGR1 (3.12–50 μM) and the control group, although high concentrations of NGR1 (100 μM) decreased cell viability (*P* < 0.05) (Figure 1B). The H9c2 cardiomyocytes were incubated with a series of AGEs concentrations (0, 50, 100, 200, and 400 μg/ml) for 12, 24, and 36 h, respectively. An MTT assay was employed to measure cell viability. Cell viability was decreased to 60.34 ± 6.52% when treated with 400 μg/ml AGEs for 36 h (Figure 1C). Thus, this study used 400 μg/mL AGEs in subsequent experiments. Subsequently, the potential cardioprotective effects of NGR1 against AGEs-induced H9c2 cardiomyocytes injury were assessed. Cell viability increased to 66.63 ± 8.34, 74.55 ± 11.74, and 80.43 ± 7.98% with pretreatment with different doses (6.25, 12.5, and 25 μM, respectively) of NGR1 for 24 h compared with exposure to only 400 μg/ml AGEs for 36 h (Figure 1D). LDH leakage, used as a biomarker of cell death, was also detected. AGEs treatment significantly increased the LDH leakage compared with the control group, and NGR1 preconditioning effectively decreased the LDH release (Figure 1E, *P* < 0.05 or *P* < 0.01). The optimal concentration and pretreatment time with NGR1 was 25 μM and 24 h, respectively. These results suggest that NGR1 could prevent H9c2 cells from AGEs-induced cell death.

### NGR1 Inhibited Mitochondrial Membrane Depolarization and Intracellular ROS Accumulation in H9c2 Cardiomyocytes Induced by AGEs

**FIGURE 2:**
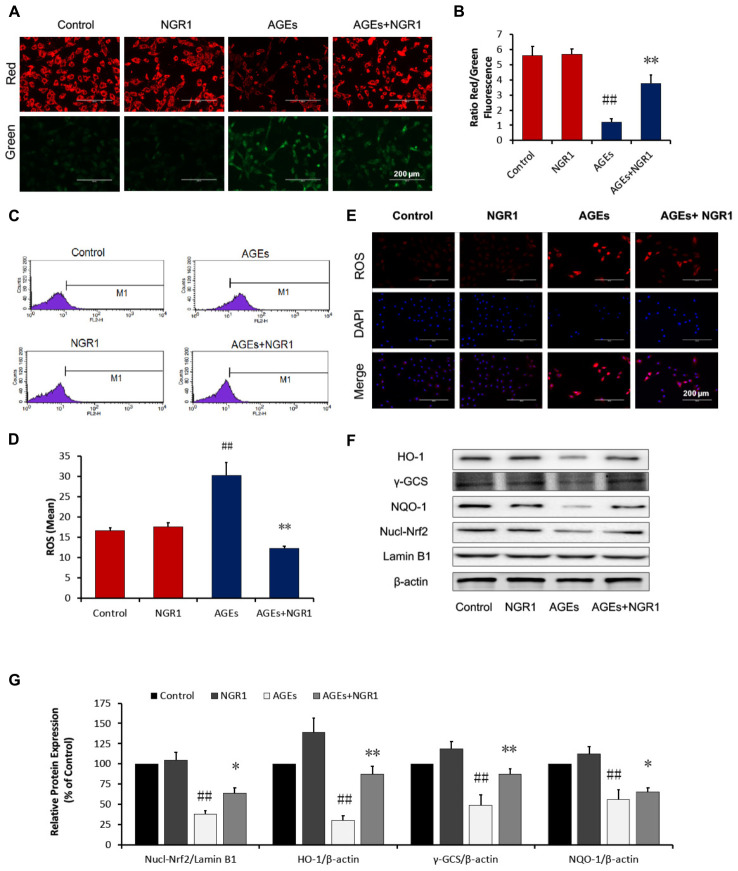
NGR1 inhibits mitochondrial membrane depolarization and intracellular ROS in H9c2 cardiomyocytes induced by AGEs. **(A,B)** Representative images and bar graphs of JC-1 red/green cells and merges showed that NGR1 increased the ratio of red to green fluorescence intensity. **(C)** Intracellular ROS levels and statistical analysis. **(D)** in H9c2 cardiomyocytes evaluated using a flow cytometer, M1 represents ROS positive cell proportion. **(E)** Representative images of MitoSOX red staining. The bar represents 200 μm. **(F)** Immunoblotting analysis of Nrf2-mediated anti-oxidative enzymes in H9c2 cells. **(G)** The relative protein expression of Nucl-Nrf2, HO-1, γ-GCS, and NQO-1 compared to β-actin are expressed in the bar graphs. The quantitative data are presented as the mean ± SD of three independent experiments. ^##^*P* < 0.01 vs. the Control group, ^∗^*P* < 0.05 or ^∗∗^*P* < 0.01 vs. the AGEs group.

JC-1 staining was used to detect the disruption of mitochondrial transmembrane potential (MTP), one of the early events of mitochondrial pathway activation of apoptosis. Mitochondria in normal H9c2 cells emitted red fluorescence after they were stained by JC-1. AGEs caused an increase in green fluorescence in H9c2 cardiomyocytes, indicating the depolarization of MTP. NGR1 preconditioning significantly inhibited depolarization of MTP and increased the red to green ratio by a wide margin (*P* < 0.01, Figures 2A,B). Oxidative stress is a key mechanism involved in the pathogenesis of cardiovascular disease in diabetes which could be induced by excess AGEs. Intracellular ROS levels were assessed by measuring mitochondrial superoxide using MitoSOX Red. AGEs treatment significantly increased ROS production in H9c2 cells by almost two-fold compared with the control. However, NGR1 preconditioning markedly attenuated this increase (Figures 2C–E, *P* < 0.01).

Many anti-oxidant genes, such as heme oxygenase-1 (HO-1) and quinine oxidoreductase 1 (NQO-1), both of which are regulated by the Nrf2 signaling, have been implicated in preventing oxidative stress. The impact of NGR1 on Nrf2-regulated antioxidant enzymes was determined. Western blot analysis showed that AGEs significantly inhibited Nrf2 translocation from the cytosol to the nucleus, and that this could be reversed by the pretreatment of H9c2 cells with NGR1 (Figures 2F,G, *P* < 0.01). Consistent with Nrf2 changes, NGR1 markedly attenuated the inhibition of HO-1, γ-GCS, and NQO-1 expression induced by AGEs (*P* < 0.01). These results collectively demonstrate that NGR1 plays a potent cardioprotective role in the inhibition of oxidative stress via the activation of Nrf2 signaling.

### NGR1 Attenuated AGEs-Induced Apoptosis in H9c2 Cardiomyocytes

**FIGURE 3:**
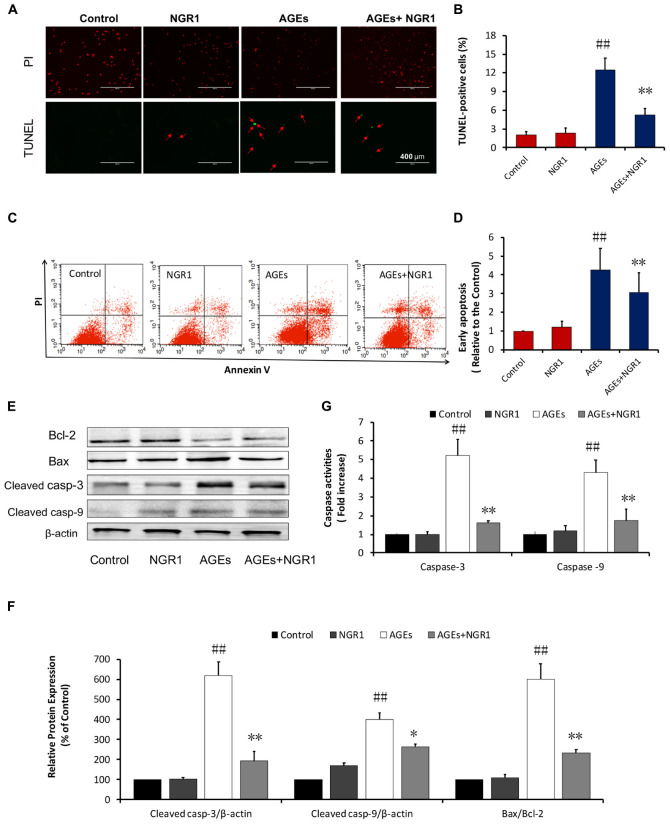
NGR1 attenuates H9c2 cell apoptosis induced by AGEs. **(A)** Representative images and **(B)** bar graphs of TUNEL-positive nuclei in green fluorescent color. The bar represents 200 μm. **(C,D)** Quantitation of flow cytometry analysis showed that NGR1 inhibited AGEs-induced H9c2 cardiomyocyte apoptosis. **(E)** The expression of apoptosis-related proteins in H9c2 cells by immunoblotting analysis. **(F)** The relative expression levels of cleaved caspase-3, cleaved caspase-9, and Bax/Bcl-2 compared to β-actin are expressed in bar graphs. **(G)** Caspase-3 and caspase-9 activation measured by fluorometric assay. The quantitative data are presented as the mean ± SD of three independent experiments. ^##^*P* < 0.01 vs. the Control group; ^∗^*P* < 0.05 or ^∗∗^*P* < 0.01 vs. the AGEs group.

Increasing evidence has shown that oxidative stress is closely related to apoptosis, which is a key factor contributing to DCM (Yu et al., 2015). A TUNEL assay was performed to examine the protective role of NGR1 in AGEs-induced H9c2 apoptosis. The percentage of TUNEL-positive cells (green) was 12.44% in the AGEs group, and 2.32% in the control group (*P* < 0.01). Pretreatment with NGR1 significantly reduced AGES-induced TUNEL labeling to 7.47% (*P* < 0.01, Figures 3A,B). Consistent with the TUNEL assay results, annexin V/PI staining and quantitative analysis by flow cytometry showed that AGEs treatment significantly increased apoptosis in H9c2 cells by almost 4.25-fold compared with the control, NGR1 preconditioning markedly reduced this increase to three-fold change (*P* < 0.01, Figures 3C,D).

Western blot analysis was further performed to assess proteins involved in the mitochondrial pathway of apoptosis. NGR1 preconditioning reversed the effects of AGEs by increasing Bcl-2 and decreasing Bax expression levels. Similarly, both cleaved caspase-3 and cleaved caspase-9 were also upregulated in H9c2 cells treated by AGEs, and their expression levels were down-regulated with NGR1 pretreatment (Figures 3E,F). Consistent with the western blotting results, caspase-3 and caspase-9 activities were significantly higher in the AGEs group than those in the control group. NGR1 showed inhibitory effects on the activation of these caspase enzymes (*P* < 0.01, Figure 3G). These results collectively indicate that NGR1 possesses anti-apoptotic effects in cardiomyocytes treated by AGEs.

### NGR1 Protects H9c2 Cardiomyocytes by Promoting Estrogen Receptor α Expression, Which Initiates Akt Signaling and Suppresses TGFβ Signaling

**FIGURE 4:**
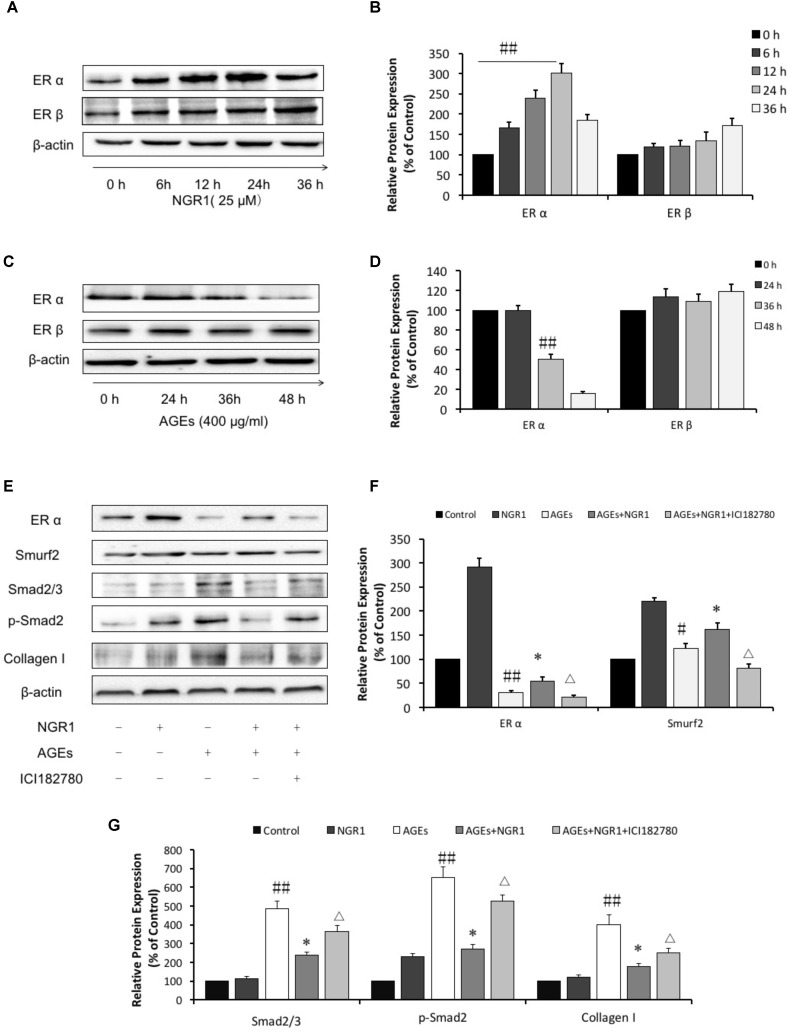
NGR1 protects H9c2 cardiomyocytes from AGEs injury by promoting the estrogen receptor expression, which subsequently initiates Akt signaling and suppresses TGFβ signaling. **(A)** The impact of NGR1 on ER protein expression. **(B)** The relative expression levels of ERα and ERβ compared to β-actin are expressed in the bar graphs. **(C)** The impact of AGEs on ER protein expression. **(D)** The relative expression levels of ERα and ERβ induced by AGEs compared to β-actin are expressed in the bar graphs. **(E)** H9c2 cells were pretreated with the ER inhibitor ICI182780 (1 μM) for 1 h, then pretreated with NGR1 for 24 h, and were subsequently incubated with AGEs for 36 h. Immunoblotting analysis determined the expression of ERα, Smurf2, Smad2/3, p-Smad2, and Collagen I. **(F)** The relative expression levels of ERα and Smurf2 compared to β-actin are expressed in the bar graphs. **(G)** The relative expression levels of Smad2/3, p-Smad2 and Collagen I compared to β-actin are expressed in the bar graphs. The quantitative data are presented as the mean ± SD of three independent experiments. ^#^*P* < 0.05 or ^##^*P* < 0.01 vs. the Control group or 0 h group, ^∗^*P* < 0.05 vs. the AGEs group, ^Δ^*P* < 0.05 vs. the AGEs + NGR1 group.

Numerous studies have shown that ER is associated with cardiovascular diseases. In addition, our previous study found NGR1 could up-regulate ER to protect against cerebral ischemia-reperfusion injury. A western blot assay showed that NGR1 significantly promoted ERα expression in a time-dependent manner. However, the impact of NGR1 on ERβ was not as significant as ERα (Figures 4A,B). Interestingly, incubation with 400 μg/mL AGEs for 36 h could markedly inhibit ERα expression (Figures 4C,D). These results collectively show that NGR1 can effectively counteract the loss of ER expression induced by AGEs.

TGFβ-mediated signaling helps to control the expression of collagens, proteins that are tightly related to cardiac hypertrophy and fibrosis. In this study we found that AGEs significantly promoted Smad2/3 and phospho-Smad2 expression, those of which played critical roles in TGFβ transduction and subsequently upregulated Collagen I (Figures 4E,G, *P* < 0.01). The increased expressions of Smad2/3, phospho-Smad2, and Collagen I activated by AGEs were reversed by pretreatment with NGR1 (*P* < 0.01), Mechanistically, AGEs suppressed Smurf2, a ubiquitinated protein regulating Smad2/3 degradation, which was reported to be associated with ERα (Ito et al., 2010). Incubation with NGR1 promisingly facilitated ERα expression and subsequently upregulated Smurf2, leading to a decrease of Smad2/3 and Collagen I, which were also attenuated by ER inhibitor (ICI182780) (Figures 4E,F, *P* < 0.01). Collectively, these results are consistent with NGR1 inhibiting TGF-beta-mediated Collagen I expression through promoting ERα expression.

### NGR1 Decreased Total Cholesterol, and Triacylglycerol Levels in db/db Mice

After 20 weeks of treatment with NGR1, FBG was determined. It was found that the impact of NGR1 on FBG was insignificant in comparison with the model group (Supplementary Figure S1A). db/db mice in the model group showed marked glucose intolerance, but the mice treated with metformin or NGR1 were partially protected from obesity-induced glucose intolerance that arises due to the lack of a leptin receptor in this animal model (Supplementary Figures S1B,C). NGR1 or metformin could inhibit body weight increase, and decrease serum TCH and TG levels in db/db mice compared with the untreated model animals (*P* < 0.05, Supplementary Figures S2A–C).

### NGR1 Prevented Diabetes-Induced Cardiac Dysfunction in T2DM Mice

**FIGURE 5:**
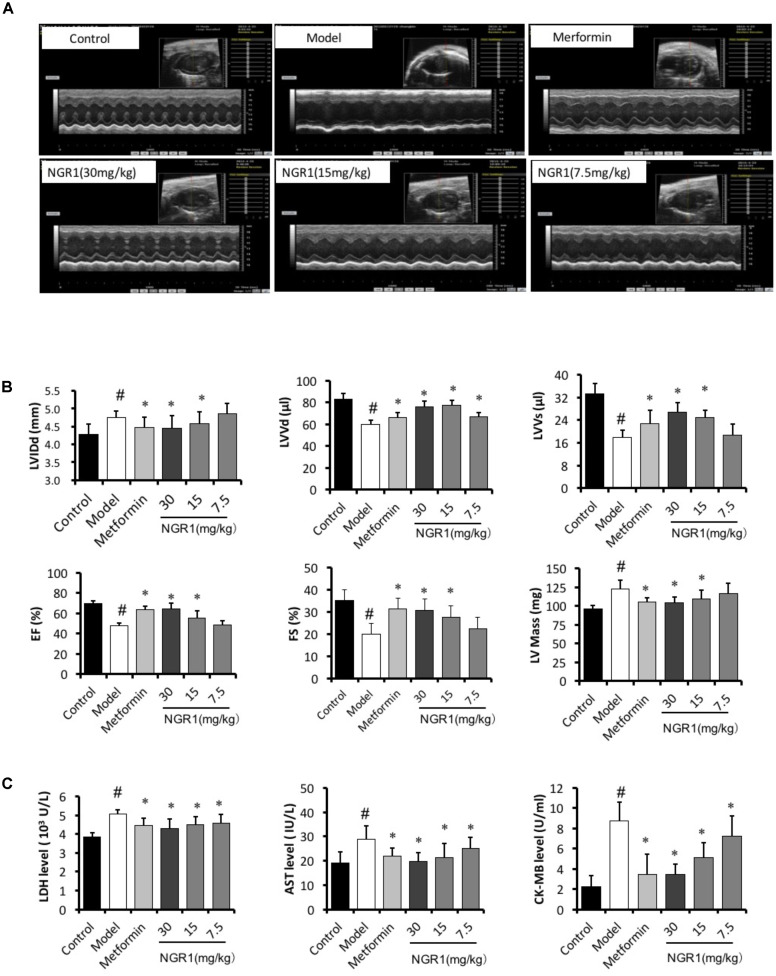
NGR1 improves cardiac function of db/db mice. **(A)** Representative image of M-mode echocardiography. **(B)** Statistics of LVIDd, LVVd, LVVs, Ejection fraction, fractional shortening, and LV mass in different groups. **(C)** NGR1 decreased serum levels of LDH, AST, and CK-MB in db/db mice. The quantitative data are presented as the mean ± SD (*n* = 8). ^#^*P* < 0.05 vs. the Control group, ^∗^*P* < 0.05 vs. the Model group.

M-mode echocardiography was performed to assess the cardiac function after 20 weeks of treatment. It was found that db/db mice had significantly increased LVIDd and LV mass, markedly decreased LVVd, LVVs, ejection fraction (EF), and fractional shortening (FS) (Figures 5A,B). Treatment of these mice with intermediate (15 mg/kg) and high (30 mg/kg) dose of NGR1 and metformin promisingly and significantly blocked the development of these cardiac dysfunctions, as evidenced by elevating EF (64.65% vs. 47.73%), FS (30.71% vs. 19.86%), LVVd (76.11 vs. 59.63 μl) and LVVs (26.8 vs. 17.98 μl).

Myocardial enzymes (LDH, CK-MB, and AST) mainly exist in cardiomyocytes. However, these enzymes are released into the serum duo to the increased permeability of cardiomyocytes induced by abnormal conditions, including myocardial ischemia, acute myocardial infarction, and cardiomyocyte apoptosis. Numerous studies have reported that serum LDH, CK-MB, and AST levels were increased in the DCM (Sun et al., 2014; Li et al., 2018). In the present study serum LDH, AST, and CK-MB levels were significantly increased in the diabetic model group compared to those of the control group, and this increase was abrogated by NGR1 treatment (*P* < 0.05, Figure 5C).

### NGR1 Prevented Diabetes-Induced Histopathological Changes in db/db Mice Through Inhibition of TGFβ-Mediated Collagen I

**FIGURE 6:**
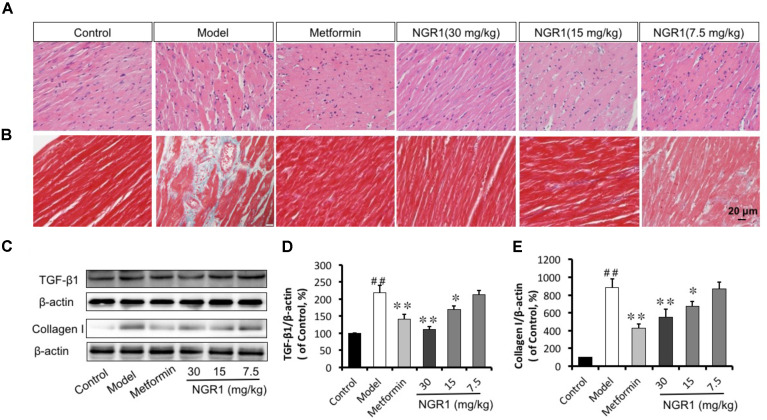
NGR1 attenuates diabetes-induced cardiac pathological alterations. **(A,B)** Reversal of diabetes-induced-cardiac hypertrophy and fibrosis by NGR1 as indicated by H&E and Masson staining (400×). **(C)** Representative images and bar graphs of TGFβ1 **(D)** and Collagen I **(E)** by western blot showed that NGR1 suppressed cardiac fibrosis. The quantitative data are presented as the mean ± SD (*n* = 3). ^##^*P* < 0.01 vs. the Control group, ^∗^*P* < 0.05 or ^∗∗^*P* < 0.01 vs. the Model group.

Hypertrophy and fibrosis play key roles in diabetes-induced cardiac remodeling. Accordingly, H&E and Masson staining were employed to assess histopathological alterations in diabetic heart. H&E staining of heart tissue showed that myocardial fibers arranged regularly and cardiac myocytes showed normal morphology in the Control group, (Figure 6A). However, in the diabetic Model group, the arrangement of cardiac fibers was disordered and the cardiac myocytes appeared swollen, which could be ameliorated by the administration of NGR1 or metformin. In addition, Masson staining showed conspicuous fibrosis in diabetic hearts, with destroyed and disorganized collagen network structure in the interstitial and perivascular areas. In contrast, the fibrotic alterations in the hearts were improved when diabetic mice were treated with NGR1 (Figure 6B). In addition, control hearts showed well-organized myofilaments, clearly visible sarcomeres, bright and dark bands, and abundant and uniform mitochondria. In contrast, diabetic hearts showed that the collagenous fiber was subject to local cytolysis, and sarcomere and mitochondria structure was irregularly arranged. These pathological features could be reversed by metformin or NGR1 treatment (Supplementary Figure S3).

Western blot analysis and immunohistochemistry were performed to assess protein levels of Collagen I as well as TGFβ1, which controls collagen production. It was found that diabetes promoted the expression of both Collagen I and TGFβ1 levels, as compared to the Control group (Figure 6C and Supplementary Figures S4A,B). Intermediate (15 mg/kg) and high (30 mg/kg) dose of NGR1 and metformin treatment significantly down-regulated the increased Collagen I and TGFβ1 expression levels in a dose-dependent manner (Figures 6D,E). Taken together, NGR1 strongly prevented TGFβ-mediated Collagen I expression, helping to attenuate diabetic hypertrophy and fibrosis.

### NGR1 Attenuated Diabetes-Induced Apoptosis in Diabetic Hearts in db/db Mice

**FIGURE 7:**
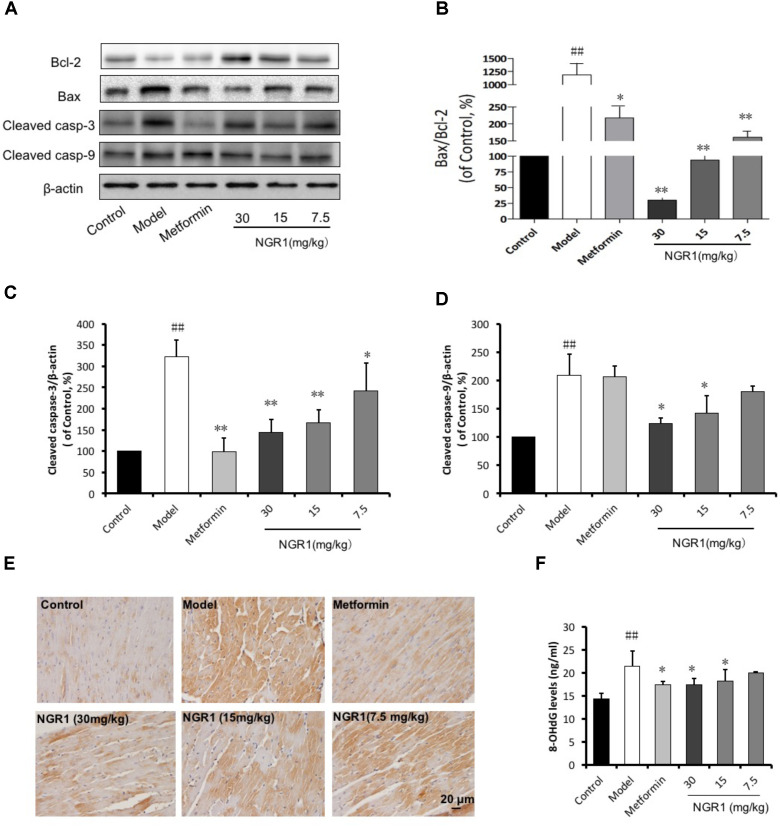
NGR1 attenuates diabetes-induced myocardial apoptosis. **(A)** Representative images of apoptotic protein immunoblots and **(B–D)** immunoblotting results quantified in bar diagrams showing that NGR1 effectively inhibited diabetes-induced apoptosis. **(E)** Representative immunohistochemical images for cleaved caspase-3. The bar represents 20 μm. **(F)** NGR1 inhibited DNA injury in db/db mice as indicated by decreasing 8-OHdG levels. The quantitative data are presented as the mean ± SD (*n* = 3). ^##^*P* < 0.01 vs. the Control group, ^∗^*P* < 0.05 or ^∗∗^*P* < 0.01 vs. the Model group.

Diabetes-induced apoptosis is highly related to cardiac structural abnormalities. Results from western blot analysis showed that the expression of the anti-apoptotic protein Bcl-2 was down-regulated, but Bax, cleaved caspase-3, and cleaved caspase-9 were up-regulated in the diabetic heart. These changes were found to be suppressed by NGR1 in a dose-dependent manner (Figures 7A–D). As well, both increased cleaved caspase-3 immunohistochemical labeling and 8-OHdG detection in diabetic mice were reduced by NGR1 treatment (Figures 7E,F). These results collectively indicated that NGR1 attenuates diabetes-induced apoptosis.

### NGR1 Promoted the Translocation of Nrf2 to the Nucleus, and Significantly Increased Antioxidant Enzymes Through Akt Signaling

**FIGURE 8:**
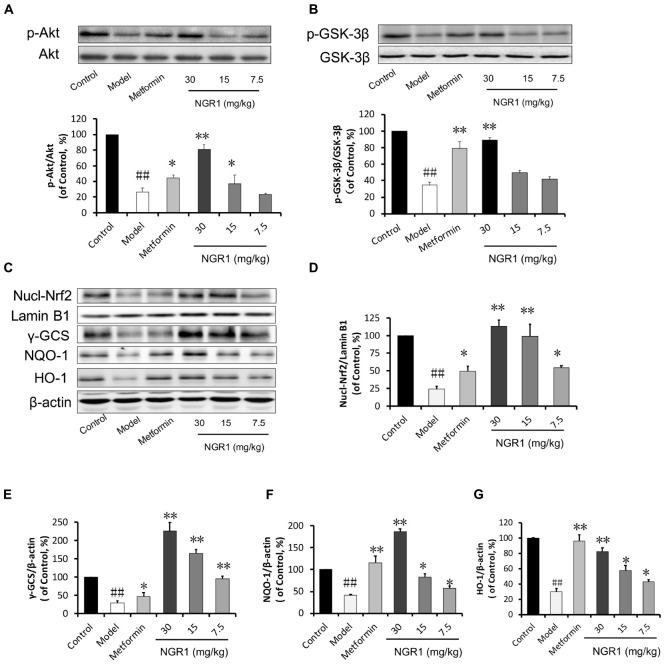
NGR1 activates the Akt-mediated Nrf2 signaling pathway. **(A,B)** Representative immunoblot images of Akt and GSK-3β, along with phospho-specific labeling for these proteins, and the immunoblotting results quantified in bar graphs showing that NGR1 promoted Akt and GSK-3β phosphorylation. **(C)** Representative immunoblot images of nuclear fractions and **(D–G)** quantification of the immunoblots shown in bar graphs of Nrf2-mediated phase II anti-oxidant enzymes (γ-GCS, NQO-1, and HO-1) showed that NGR1 promoted the translocation of Nrf2 to the nucleus. This was associated with the up-regulation of these anti-oxidant enzymes. The quantitative data are presented as the mean ± SD (*n* = 3). ^##^*P* < 0.01 vs. the Control group, ^∗^*P* < 0.05 or ^∗∗^*P* < 0.01 vs. the Model group.

Given that NGR1 could attenuate diabetes-associated apoptosis, we hypothesized that NGR1 regulated antioxidant enzymes, which are main contributors in fighting against oxidative stress. Western blot results showed that the translocation of Nrf2 and γ-GCS, NQO-1, and HO-1 expressions were suppressed in diabetic mice in comparison with the control group (Figures 8C–G). Also, results from immunohistochemical staining showed that HO-1 in diabetic heart was inhibited (Supplementary Figure S4C). These pathological alterations were reversed by NGR1 treatment in a dose-dependent manner (Figures 8C–G). The PI3K/Akt pathway is involved in multiple cellular processes and it is reported to signal upstream of Nrf2 (Lee and Jeong, 2016). We found that phosphorylation of Akt was significantly inhibited in diabetic hearts (0.25-fold of control, *P* < 0.01, Figure 8A). The phosphorylation of GSK-3β located downstream of Akt was also inhibited in diabetic hearts, and was tightly related with the degradation of Nrf2 (Figure 8B). NGR1 or metformin treatments were found to enhance the phosphorylation of Akt and GSK-3β, thereby exerting anti-oxidative effects in the protection against DCM.

### NGR1 Protected db/db Mice From Diabetic Cardiomyopathy via Estrogen Receptor α-Dependent Up-Regulation of Smurf2 Expression

**FIGURE 9:**
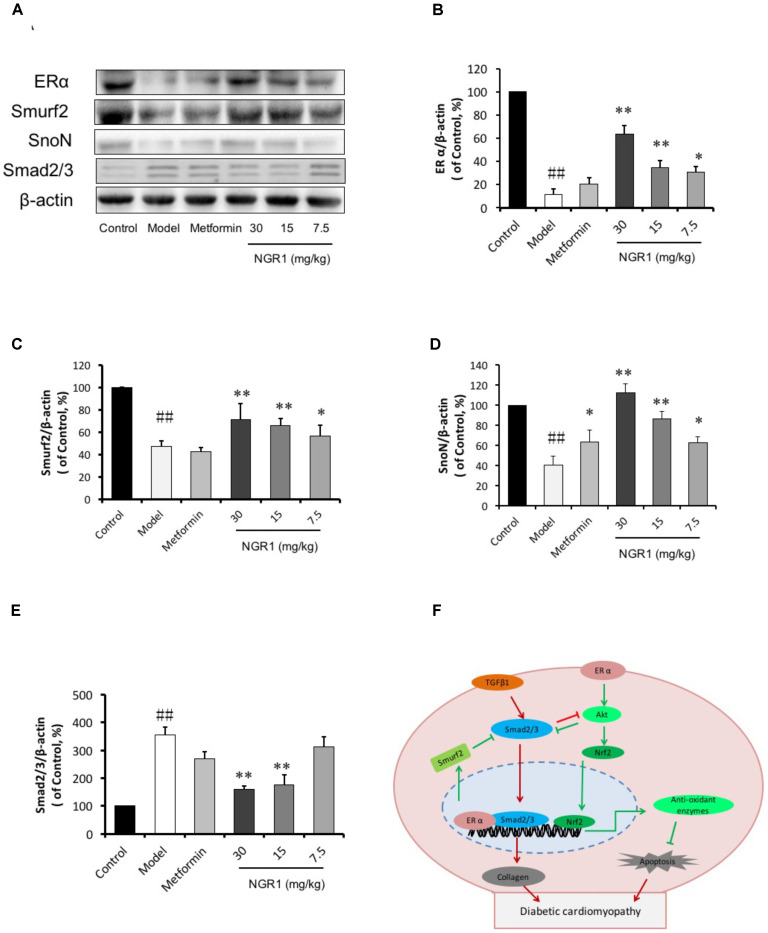
NGR1 inhibits Smad2/3 expression by promoting Smurf2 expression in an estrogen receptor α-dependent manner. **(A)** Representative images of ERα, Smurf2, SnoN, and Smad2/3 protein expression determined by western blot. **(B–E)** The relative expression levels of ERα, Smurf2, SnoN, and Smad2/3 compared to β-actin are expressed in the bar graphs. The quantitative data are presented as the mean ± SD (*n* = 3). ^##^*P* < 0.01 vs. the Control group, ^∗^*P* < 0.05 or ^∗∗^*P* < 0.01 vs. the Model group. **(F)** Schematic illustration showing the mechanisms underlying the prevention of diabetes/AGEs- induced injury in cardiomyocytes and db/db mice by NGR1. ERα, estrogen receptorα; TGF-β, transforming growth factor β; Smurf2, Smad-mediated ubiquitination regulatory factor 2; Nrf2, nuclear factor erythroid 2-related factor 2; Akt, protein kinase B.

We have previously reported that NGR1 elevates estrogen receptor levels to subsequently up-regulate Akt and Nrf2-mediated HO-1 (Meng et al., 2014). We have now found that ERα in diabetic hearts was highly reduced (0.11-fold of control, *P* < 0.01, Figures 9A,B). Of note, Smurf2, a ubiquitinated protein reported to interact with ERα, was also down-regulated in the diabetic model group (Figures 9A,C). In addition, the expression of SnoN, another regulator of Smad2/3 was found to decrease (Figures 9A,D). In contrast, the phosphorylation of Smad2/3 increased in the diabetic hearts. NGR1 treatment was found to enhance the expression of ER and Smurf2 and to limit the phosphorylation of Smad2/3, demonstrating beneficial mechanisms relevant to the treatment of DCM (Figure 9F).

## Discussion

In the present study, we have demonstrated, for the first time, that treatment with NGR1 could protect the diabetic heart from the structural and functional abnormalities that are associated with persistent oxidative stress and apoptosis in db/db mice. Mechanistically, NGR1 activates estrogen receptor α, which up-regulates Nrf2-induced phase II anti-oxidative enzymes in an Akt-dependent manner (Figure 9F). Additionally, estrogen receptor α induction by NGR1 could promote Smurf2 expression. This plays a pivotal role in the inhibition of TGFβ signaling, which is associated with cardiac hypertrophy and fibrosis. These findings reveal that NGR1 might be a potential therapeutic agent to prevent DCM.

In the past decades, great progress has been achieved in the treatment of diabetes complications. Increasing evidence has demonstrated that DCM is correlated with superfluous AGEs owing to abnormal metabolic function in diabetes. Overproduction of AGEs in diabetes is a risk factor for DCM (Yuan et al., 2014; Guo et al., 2015; Hou et al., 2016), which leads to severe oxidative stress and inflammatory responses. Ko et al. (2015) showed that 400 μg/ml AGEs could increase ROS production through inhibition of antioxidant Nrf-2 and its downstream pathway in H9c2 cells. AGEs are reported to increase ROS production by RAGE/TLR4-NF-κB-ROS pathways (Ohtsu et al., 2017) and hyperglycemia stimulates ROS generation via activation of MAPK and PKC-NAD(P)H oxidase pathway (Batchuluun et al., 2014; Cheng Y.S. et al., 2016). Thus, AGEs were employed for *in vitro* experiments to induce H9c2 cardiomyocyte injury. This injury was attenuated by pretreatment with 25 μM NGR1, as indicated by a decrease in LDH release. The mechanism that causes cardiac injury by AGEs is reported to be associated with detrimental endoplasmic reticulum stress, inflammation, oxidative stress and mitochondrial damage (Yang et al., 2015; Zhao et al., 2016; Ishibashi et al., 2017). Consistent with previous literature, in our study, AGEs were found to strongly inhibit H9c2 cell viability accompanied by aggressive ROS and massive apoptosis both *in vitro* (Figures 2, 3), and *in vivo* (Figure 7).

Diabetic cardiomyopathy is more closely associated with type 2 DM (T2DM) compared with type 1 DM, with an early hallmark being left ventricular (LV) diastolic dysfunction preceding the onset of systolic dysfunction (Schannwell et al., 2002). To date the pathogenesis of LV dysfunction in diabetes is still poorly understood. The transgenic db/db mice in this work were utilized to establish the development of DCM in the T2DM model, in which the diabetic hearts exhibited morphological alterations, increased interstitial collagen deposition, and up-regulation of TGF-β1, illustrating evident cardiac fibrosis and hypertrophy (Figure 6). Additionally, db/db mice were examined by M-mode echocardiography and showed impaired cardiac function including increased LVIDd and LV mass, decreased LVVd, LVVs, EF, and FS (Figures 5A,B). These changes were ameliorated by NGR1 and metformin treatment. Although NGR1 showed no apparent effect on the extent of hyperglycemia, NGR1 did show similar or enhanced cardiac protective effect relative to metformin. Key to this may be the mechanistic differences between these two agents. Metformin activates AMPK subsequently ameliorating hyperglycemia (Zhou Z. et al., 2016) and inflammation (Zhou Z.Y. et al., 2016). In comparison, NGR1 not only reduced oxidative stress through Akt-dependent activation of Nrf2 signaling, but also significantly suppressed TGF-β1 signaling that mediates expression of collagen I and is tightly related to cardiac fibrosis.

The estrogen receptor has been reported to be involved in multiple diseases, such as breast cancer, fatty liver, endometriosis and metabolic disease (Ribas et al., 2016; Besse-Patin et al., 2017; Magnani et al., 2017). To our interest, the estrogen receptor also plays pivotal roles in the cardiovascular system, promoting vasorelaxation, reducing low-density lipoprotein cholesterol level and protecting against cardiac hypertrophy and ischemia-reperfusion (I/R) (Skavdahl et al., 2005; Babiker et al., 2014). Indeed, it was found in this study that superfluous AGEs could markedly inhibit ERα expression (Figures 4C,D, *P* < 0.01). Interestingly, with the treatment of NGR1, ERα was found to be upregulated in a time-dependent manner, but another receptor, ERβ, was insignificantly regulated within 24 h, indicating that the upregulation of ERα by NGR1 was more relevant than the latter in our model, which was in accordance with our previous research (Sun et al., 2013). With NGR1 treatment serum TCH and TG levels in db/db mice significantly decreased. NGR1 promoted lipid metabolism in an ERα-dependent pathway. Importantly, ERα plays a role in reverse cholesterol transport and its deletion causes increased adiposity and enhanced insulin resistance (Zhu et al., 2018). Thus, NGR1 may upregulate ERα expression to accelerate TCH and TG metabolism, which could supply energy for the heart. Meanwhile NGR1 reduced lipotoxicity induced by accumulation of TCH and TG in heart.

Given that impaired ER action impacts on mitochondrial function and metabolic homeostasis, promoting obesity and metabolic dysfunction, we decided to investigate the mechanism by which estrogen receptor protected against DCM with NGR1. ERα is located both at the plasma membrane (Razandi et al., 2003) and the nucleus (Kim et al., 2016), and participates in various signaling pathways including PI3K/Akt, MAPK, and Wnt4 signaling (Klinge et al., 2005; Miyakoshi et al., 2009). Consistent with previous research, NGR1 possessed the ability to activate Akt in an ERα-dependent manner, which subsequently promoted Nrf2 translocation to nucleus to up-regulate phase II antioxidant enzymes including HO-1, NQO-1, and γ-GCS both *in vitro* (Figures 2F,G) and *in vivo* (Figure 7). The antioxidant enzymes up-regulated by NGR1 could ameliorate AGEs and/or hyperglycemia- induced oxidative stress, as indicated by NGR1’s limiting of mitochondrial membrane depolarization (mtPTP opening) and inhibition of intracellular ROS accumulation in H9c2 cardiomyocytes. Apoptosis, being tightly associated with aggressive oxidative stress, is considered to contribute to the development of DCM. AGEs or hyperglycemia in diabetic db/db mice can increase the Bax/Bcl-2 ratio by generating superfluous ROS, which can decrease mtPTP and subsequently activate caspase-3. Treatment with NGR1 or metformin decreased the expression of pro-apoptotic Bax, and increased expression of the anti-apoptotic Bcl-2, suggesting that the inhibition of cardiac cell apoptosis may be one of the important mechanisms by which NGR1 fights against DCM.

TGFβ signaling is involved in fibrosis and hypertrophy (Biernacka et al., 2015), and TGFβ1 promotes Smad2/3 phosphorylation and translocation to the nucleus, which in turn increases collagen production. This collagen then accumulates in the extracellular matrix. Consistent with other reports, the protein levels of TGF-β1, Smad2/3 (Figure 9E), and Collagen I in diabetic hearts is increased, and these increases could be reversed by NGR1 or metformin administration. These results collectively substantiate that NGR1 can limit cardiac fibrosis and hypertrophy in db/db mice via the suppression of TGFβ signaling.

The TGFβ pathway is capable of regulating multiple cellular responses; thus, an exact control of this signaling is essential. As previously reported, inhibitory Smad7 (I-Smad-7), and SnoN (c-Ski) are two endogenous inhibitors that antagonize TGFβ signaling (Kavsak et al., 2000). Smad-mediated ubiquitination regulatory factor 2 (Smurf2) recruited by I-Smad-7 targets the type I TGFβ receptor for degradation to inhibit this pathway (Cao et al., 2014; Malonis et al., 2017). Accordingly, Smurf2 interacts with Smad7 to suppress TGFβ-mediated fibrosis, including liver fibrosis and tubulointerstitial fibrosis (Liu et al., 2007; Cai et al., 2012). Thus, upregulation of Smurf2 levels may be an effective strategy to inhibit TGFβ signaling.

A number of studies have shown NGR1 to be a phytoestrogen capable of protecting against cardiovascular disease via upregulation of the estrogen receptor (Sun et al., 2013; Zhong et al., 2015). Consistent with these studies, NGR1 increased estrogen receptor alpha expression both in H9c2 cells and diabetic heart to exert its protective roles. The increased estrogen receptor alpha promoted Smurf2 expression and subsequently formed a ternary complex with both Smurf2 and Smad to inhibit TGF-β pathways (Ito et al., 2010). In the present study we have shown decreased levels of Smurf2 in both the intact diabetic heart and H9c2 cells exposed to AGEs. In both cases this was reversed by NGR1. In the situation of estrogen receptor inhibition (with ICI182780) Smurf2 activation was prevented and ERα was down-regulated. Collectively, these observations are consistent with NGR1 promoting Smurf2 expression causing Smad2/3 degradation and reduced TGFβ-mediated collagen production.

The current studies provide some initial insight into a role for NGR1 in regulation of ERα in db/db mice and H9c2 cardiomyocytes. How the ERα interacts with Smurf2 or SnoN was not verified in this work. Furthermore, to better illuminate the exact mechanisms by which ERα inhibits DCM, it would be intriguing to utilize an ERα-conditional knockout mouse model. The protective effects of NGR1 in ERα-knockout H9c2 cardiomyocytes are unknown. The observation that NGR1 treatment decreases TG and TCH is suggestive that this agent may inhibit lipotoxicity and thereby improve DCM. Thus it may be fruitful, in future studies, to explore the impact of NGR1 on abnormalities of cardiac lipid metabolism and palmitate-induced H9c2 cell injury.

## Conclusion

The results of the present work demonstrate that NGR1 possesses the capacity to protect the diabetic heart from structural and functional abnormalities through the activation of Akt-dependent Nrf2 signaling and inhibition of the TGFβ pathway in an estrogen receptor α-dependent manner. Accordingly, NGR1 may provide a therapeutic approach for protecting against diabetes-induced cardiomyopathy.

## Author Contributions

GS and XS conducted the study. BZ and JZ designed the detailed experiments, performed the study, and collected and analyzed data. CZ, XZ, and JY took part in the animal experiments in this study. BZ wrote the manuscript and SK helped to revise it. All authors discussed, edited, and approved the final version.

## Conflict of Interest Statement

The authors declare that the research was conducted in the absence of any commercial or financial relationships that could be construed as a potential conflict of interest.
