# Corrigendum: Notoginsenoside R1 protects against diabetic cardiomyopathy through activating estrogen receptor α and its downstream signaling

**DOI:** 10.3389/fphar.2024.1488083

**Published:** 2025-01-22

**Authors:** Bin Zhang, Jingyi Zhang, Chenyang Zhang, Xuelian Zhang, Jingxue Ye, Shihuan Kuang, Guibo Sun, Xiaobo Sun

**Affiliations:** ^1^ Institute of Medicinal Plant Development, Peking Union Medical College and Chinese Academy of Medical Sciences, Beijing, China; ^2^ Key Laboratory of Bioactive Substances and Resources Utilization of Chinese Herbal Medicine, Ministry of Education, Beijing, China; ^3^ Beijing Key Laboratory of Innovative Drug Discovery of Traditional Chinese Medicine (Natural Medicine) and Translational Medicine, Beijing, China; ^4^ Key Laboratory of Efficacy Evaluation of Chinese Medicine Against Glyeolipid Metabolism Disorder Disease, State Administration of Traditional Chinese Medicine, Beijing, China; ^5^ Department of Animal Sciences, Purdue University, West Lafayette, IN, United States

**Keywords:** diabetes mellitus, diabetic cardiomyopathy, estrogen receptor, apoptosis, oxidative stress

In the published article, there was an error in Figure 2 as published. Specifically, Western blot pictures of HO-1and NQO-1 in Figure 2F were misplaced. After checking the raw data, the misplaced Western blot pictures of HO-1and NQO-1 were corrected. The corrected Figure 2 and its caption appears below.

**FIGURE 2:**
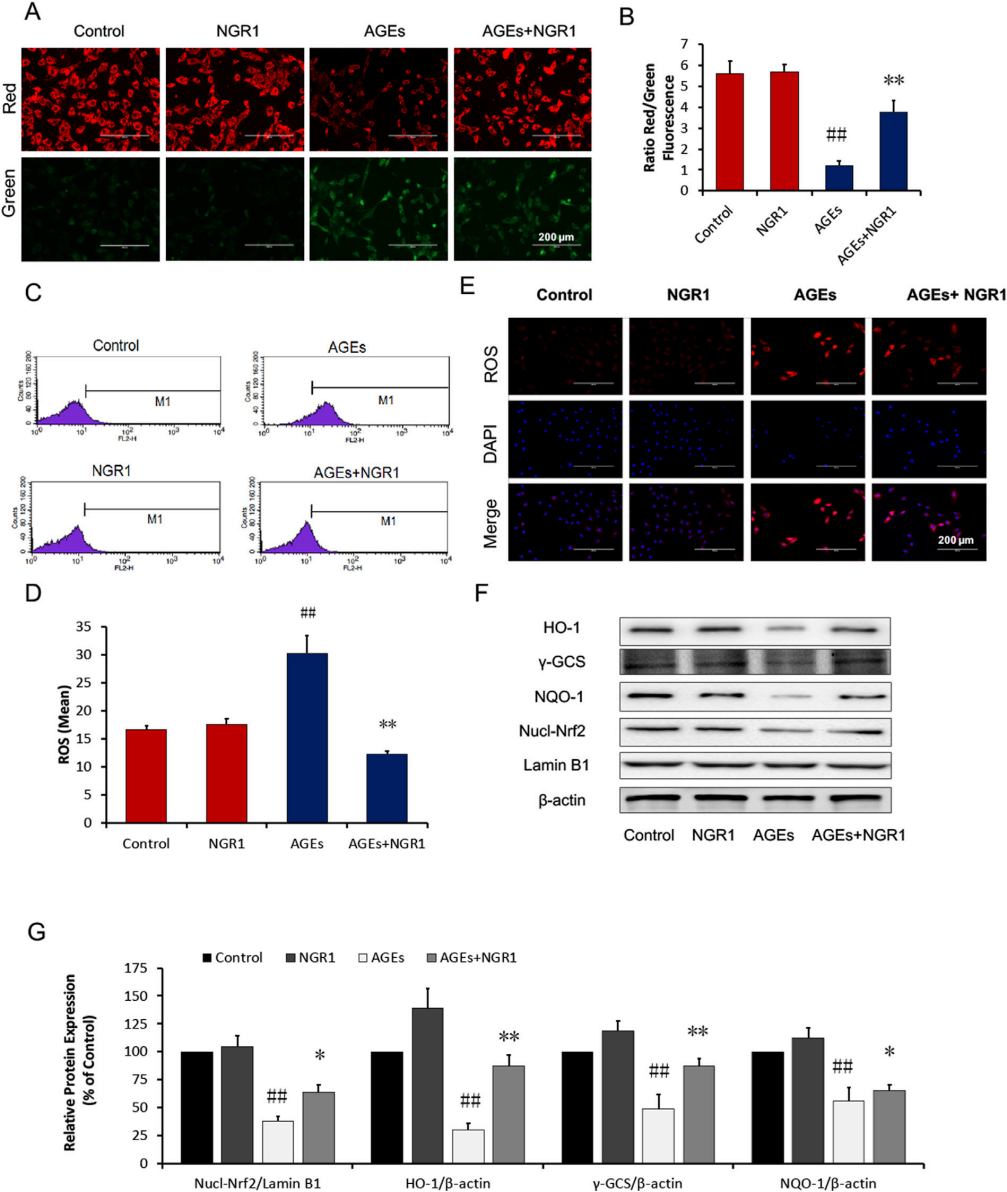
NGR1 inhibits mitochondrial membrane depolarization and intracellular ROS in H9c2 cardiomyocytes induced by AGEs. **(A, B)** Representative images and bar graphs of JC-1 red/green cells and merges showed that NGR1 increased the ratio of red to green fluorescence intensity. **(C)** Intracellular ROS levels and statistical analysis. **(D)** in H9c2 cardiomyocytes evaluated using a flow cytometer, M1 represents ROS positive cell proportion. **(E)** Representative images of MitoSOX red staining. The bar represents 200 μm. **(F)** Immunoblotting analysis of Nrf2-mediated anti-oxidative enzymes in H9c2 cells. **(G)** The relative protein expression of Nucl-Nrf2, HO-1, γ-GCS, and NQO-1 compared to β-actin are expressed in the bar graphs. The quantitative data are presented as the mean ± SD of three independent experiments. ^##^ *P* < 0.01 vs. the Control group, *P < 0.05 or **P < 0.01 vs. the AGEs group.

In the published article, there was an error in Figure 3 as published. The TUNEL staining picture in AGEs group was misplaced in Figure 3A. Additionally, the Western blot pictures of Bcl-2 and cleaved casp-3 were misplaced in Figure 3E. After checking the raw data, the misplaced bands of Bcl-2 and cleaved casp-3 were corrected. The corrected Figure 3 and its caption appears below.

**FIGURE 3:**
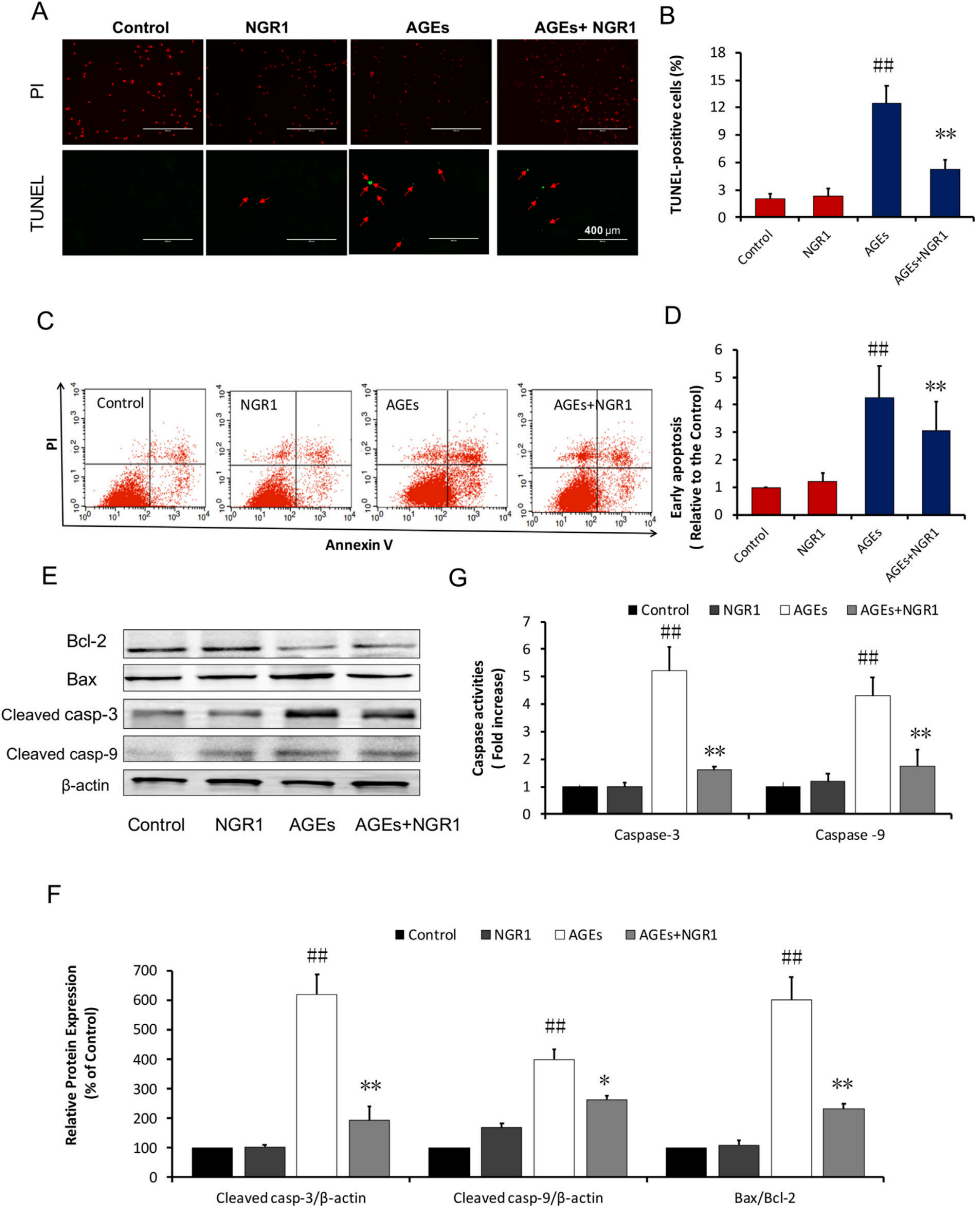
NGR1 attenuates H9c2 cell apoptosis induced by AGEs. **(A)** Representative images and **(B)** bar graphs of TUNEL-positive nuclei in green fluorescent color. The bar represents 200 μm. **(C, D)** Quantitation of flow cytometry analysis showed that NGR1 inhibited AGEs-induced H9c2 cardiomyocyte apoptosis. **(E)** The expression of apoptosis-related proteins in H9c2 cells by immunoblotting analysis. **(F)** The relative expression levels of cleaved caspase-3, cleaved caspase-9, and Bax/Bcl-2 compared to β-actin are expressed in bar graphs. **(G)** Caspase-3 and caspase-9 activation measured by fluorometric assay. The quantitative data are presented as the mean ± SD of three independent experiments. ^##^ *P* < 0.01 vs. the Control group; *P < 0.05 or **P < 0.01 vs. the AGEs group.

In the published article, there was an error in Figure 8 as published. The Lamin B1 and β-actin bands in Figure 8C were misplaced. After checking the raw data, the misplaced bands were corrected. The corrected Figure 8 and its caption appears below.

**FIGURE 8:**
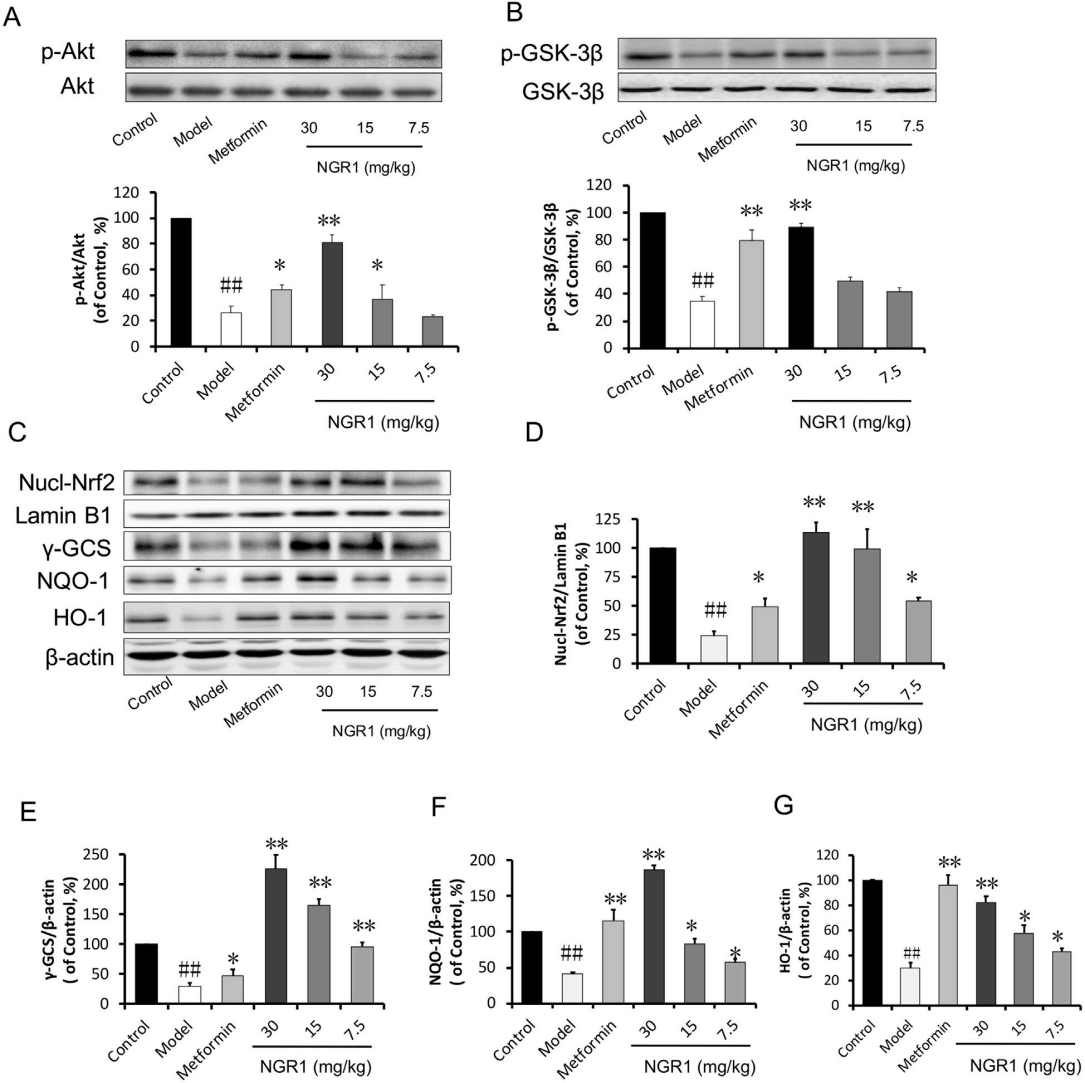
NGR1 activates the Akt-mediated Nrf2 signaling pathway. **(A, B)** Representative immunoblot images of Akt and GSK-3β, along with phospho-specific labeling for these proteins, and the immunoblotting results quantified in bar graphs showing that NGR1 promoted Akt and GSK-3β phosphorylation. **(C)** Representative immunoblot images of nuclear fractions and **(D–G)** quantification of the immunoblots shown in bar graphs of Nrf2-mediated phase II anti-oxidant enzymes (γ-GCS, NQO-1, and HO-1) showed that NGR1 promoted the translocation of Nrf2 to the nucleus. This was associated with the upregulation of these anti-oxidant enzymes. The quantitative data are presented as the mean ± SD (*n* = 3). ^##^ *P* < 0.01 vs. the Control group, *P < 0.05 or **P < 0.01 vs. the Model group.

The authors apologize for these errors and state that this does not change the scientific conclusions of the article in any way. The original article has been updated.

