# Supplementary material for: Notoginsenoside R1 Protects Against Diabetic Cardiomyopathy Through Activating Estrogen Receptor α and Its Downstream Signaling
Source: Front Pharmacol. 2018 Nov 2;9:1227. doi: 10.3389/fphar.2018.01227 (PMC6224485; doi:10.3389/fphar.2018.01227)
Supplement: Supplementary file 2 [file Presentation_1.pdf]

Supplementary Figures Legends.

FIGURE S1 NGR1 does not affect fasting blood glucose levels in db/db mice, but improves glucose tolerance.

(A) Fasting blood glucose level in db/db mice with NGR1 and metformin treatment. **(B, C)** Glucose tolerance test (GTT) and area under the curve (AUC) evaluation. The quantitative data are presented as the mean ± SD (n=8). $^{\#\#}$ $P < 0.01$ vs. the Control group, * $P < 0.05$ or ** $P < 0.01$ vs. the Model group.

FIGURE S2 NGR1 slows body weight gain and decreases serum lipid levels in db/db mice.

(A) Body weight changes in db/db mice with NGR1 and metformin administration. **(B, C)** NGR1 reduced triglycerides (TG) and total cholesterol (TCH) levels in db/db mice. The quantitative data are presented as the mean ± SD (n=8). * $P < 0.05$ or ** $P < 0.01$ vs. the Model group.

FIGURE S3 NGR1 ameliorates myocardial ultrastructure injury.

Representative TEM images from different groups. Control hearts showed well-organized myofilaments, and abundant and uniform mitochondria. In contrast, diabetic hearts displayed that the collagenous fiber was subject to local cytolysis, and sarcomere and mitochondria structure was irregularly arranged. These pathological features could be reversed by metformin or NGR1 treatment.

FIGURE S4 Representative immunohistochemical images for TGF-β1 (A), Collagen I (B), and HO-1 (C). The bar represents 20 μm.

TABLE S1 Primary anti-bodies and second anti-bodies used in this study are listed in the table.
