# Supplementary figures and images for: Notoginsenoside R1 Protects Against Diabetic Cardiomyopathy Through Activating Estrogen Receptor α and Its Downstream Signaling
Source: Front Pharmacol. 2018 Nov 2;9:1227. doi: 10.3389/fphar.2018.01227 (PMC6224485; doi:10.3389/fphar.2018.01227)

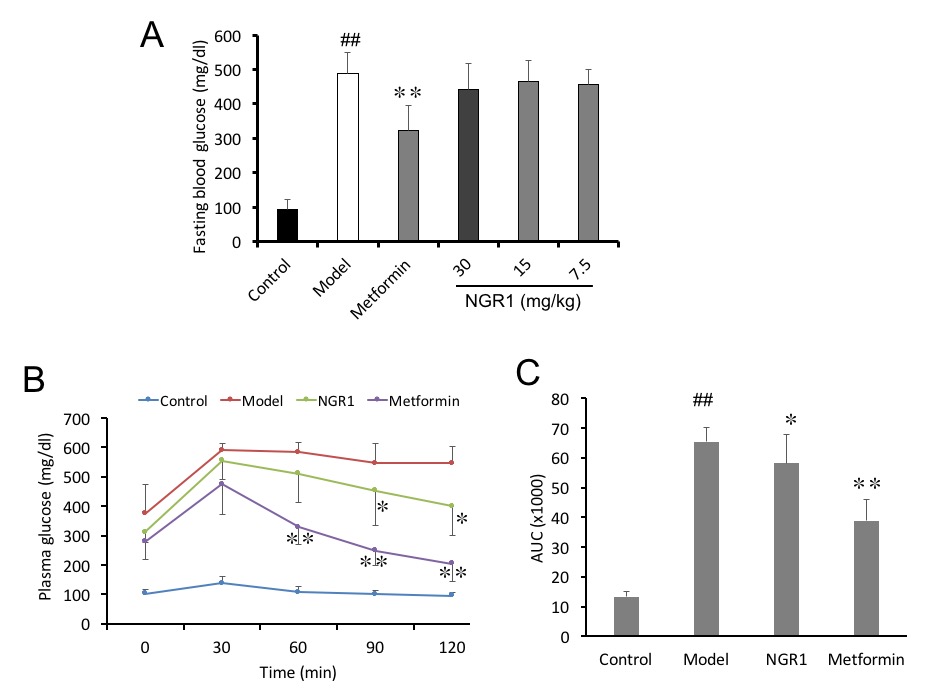

Supplement: Supplementary file 3 [file Image_1.JPEG]

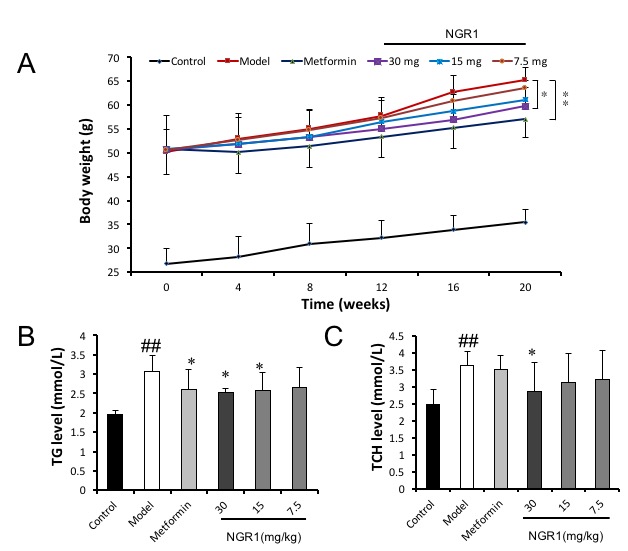

Supplement: Supplementary file 4 [file Image_2.JPEG]

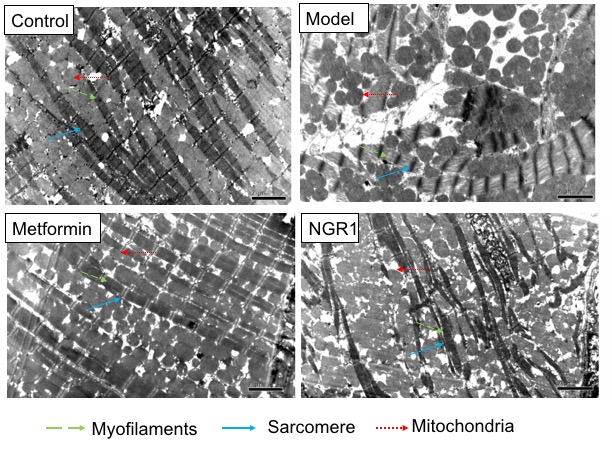

Supplement: Supplementary file 5 [file Image_3.JPEG]

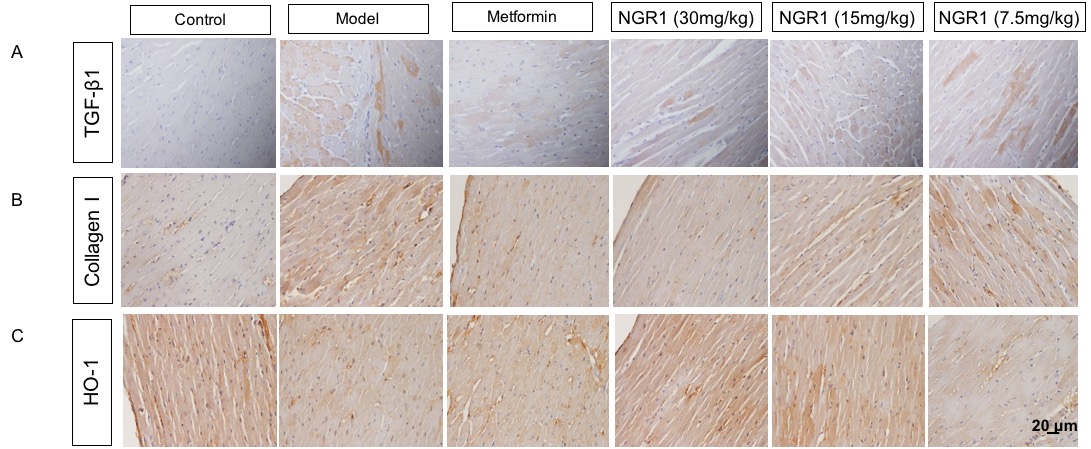

Supplement: Supplementary file 6 [file Image_4.JPEG]
